# Integrating Transcriptomics and Metabolomics to Comprehensively Analyze Phytohormone Regulatory Mechanisms in *Rhododendron chrysanthum* Pall. Under UV-B Radiation

**DOI:** 10.3390/ijms26041545

**Published:** 2025-02-12

**Authors:** Wang Yu, Qi Sun, Hongwei Xu, Xiaofu Zhou

**Affiliations:** Jilin Provincial Key Laboratory of Plant Resource Science and Green Production, Jilin Normal University, Siping 136000, China

**Keywords:** UV-B, hormone pathways, *Rhododendron chrysanthum* Pall., metabolomics, transcriptome

## Abstract

In order to fully elucidate the roles and systems of phytohormones in UV-B radiation (UV-B) leaves of the *Rhododendron chrysanthum* Pall. (*R. chrysanthum*), we conducted a comprehensive analysis of how *R. chrysanthum* protects itself against UV-B using transcriptomic and metabolomic data. Transcript and metabolite profiles were generated by a combination of deep sequencing and LC-MS/MS (liquid chromatography–tandem mass spectrometry), respectively. Combined with physiological and biochemical assays, we studied compound accumulation, biosynthesis and expression of signaling genes of seven hormones and the effects of hormones on plant photosynthesis. The findings indicate that during leaf defense against UV-B, photosynthesis declined, the photosynthetic system was impaired and the concentration of salicylic acid (SA) hormones increased, whereas the contents of cytokinin (CK), abscisic acid (ABA), ethylene, auxin, jasmonic acid (JA) and gibberellins (GAs) continued to decrease. Finally, correlation tests between hormone content and genes were analyzed, and genes closely related to leaf resistance to UV-B were identified in seven pathways. These results will expand our understanding of the hormonal regulatory mechanisms of plant resistance to UV-B and at the same time lay the foundation for plant resistance to adversity stress.

## 1. Introduction

Light is an important environmental signal on which plants depend for survival; however, it is also a source of abiotic stress for plants [1,2,3]. UV-B radiation (UV-B) (280–315 nm) light is an intrinsic part of the sunlight that reaches the Earth’s surface. Plants serve as sequestering organisms, and therefore, exposure to UV-B throughout their life cycle is unavoidable. Although the atmospheric ozone layer absorbs most of the UV-B, about 5% of solar UV-B reaches the Earth’s surface [4,5]. UV-B can be potentially harmful to plants. It not only impairs the photosynthetic system of plants but also reduces chlorophyll content, causes DNA damage and affects DNA replication and transcription, thereby inhibiting plant development and metabolism [6,7,8].

Hormones have a major regulatory role in plant growth, development and adaptation to the environment. When plants defend themselves against abiotic stresses, multiple hormones cross-talk and co-regulate. Each hormone has its own unique function in the signal transduction process, and the mechanism of action for regulating plant stress tolerance is also different [9,10]. Low-temperature stress induces the production of abscisic acid (ABA) to activate ABA-dependent signaling response pathways and increase ABA content in plants, thereby positively regulating plant tolerance to adversity stress [11,12]. Abiotic stress can induce the expression of genes related to jasmonic acid (JA) synthesis in response to various unfavorable conditions [13]. Salicylic acid is involved in regulating plant response to UV-B stress, and SA serves as a signal to reduce oxidative stress by triggering the up-regulation of antioxidants, thereby promoting growth and photosynthesis [14]. It was found that cytokinin (CK) can cross-talk with ethylene signaling to co-regulate plant response to low-temperature signaling [15]. It has been shown that cold stress causes GA-induced degradation of *DELLA* deterrent proteins in *Arabidopsis thaliana*, which controls many key developmental processes and responses to stresses such as cold [16]. Therefore, the study of changes in hormone levels in plants as well as phytohormone signaling networks is essential for understanding plant responses to various types of environmental stresses.

*Rhododendron chrysanthum* Pall. (*R. chrysanthum*) is a rhododendron growing in high-altitude and low-temperature mountainous areas, which is one of the rare germplasm resources in the world [17]. After a long adaptive evolutionary process, *R. chrysanthum* has evolved resistance to abiotic stresses such as low temperature, drought and strong UV-B, and thus can be used as a good experimental material for exploring plants’ resistance to UV-B [18]. Existing studies on *R. chrysanthum* have focused on the response of its metabolic pathways to UV-B [19]. Hormonal studies on *R. chrysanthum*, on the other hand, have focused on the role played by exogenous ABA in regulating the metabolic pathways of *R. chrysanthum* [20]. Meanwhile, the complete study of changes in various phytohormones, their biosynthesis, and signaling-related genes and metabolites in *R. chrysanthum* under UV-B stress is less studied.

Not only are individual metabolic pathways regulated by environmental factors, but the homeostasis of the entire metabolic network is also affected [21]. Compared to traditional physiological and biochemical studies and genetic phenotyping, histological research techniques allow for a more systematic observation of physiological changes in plants and may offer greater potential for the discovery of new genes [22,23]. In recent years, the association analysis of various organizational platforms has emerged as a new trend in the field of plant metabolism research [24,25]. In this experiment, seven important hormones were studied transcriptomically and metabolomically in *R. chrysanthum*.

The present study is a comprehensive study of various types of phytohormones that have been detected in *R. chrysanthum*, from biosynthesis to signaling, based on previous studies [26,27]. Changes in metabolite levels and pathway gene expression levels during the defense of *R. chrysanthum* against UV-B demonstrated the mechanisms by which different hormones regulate plant leaves against abiotic stresses. This is the first complete transcriptomic and metabolomic analysis of phytohormonal regulation during the defense of *R. chrysanthum* against UV-B radiation. The findings enrich existing insights into the mechanisms of phytohormone action and provide new perspectives in the field of phytohormone response to UV-B.

## 2. Results

### 2.1. Differential Photosynthetic Performance upon UV-B Exposure

By comparing the photosynthetic characteristics of UV-B radiation in *R. chrysanthum*, such as Fv/Fm and Fm, the effect of UV-B radiation on *R. chrysanthum* was observed. The fluorescence of chlorophyll was measured by IMAGING PAM. The results showed that Fv/Fm and Fm decreased significantly after UV-B radiation (Supplementary Figure S1).

In order to further understand the changes in the electron transport chain of the photosynthetic system without leaves under the same treatment, five JIP assay parameters were selected for reflecting the changes in the activity of the electron transport chain. As shown in Supplementary Figure S2, UV-B radiation significantly reduced the performance index of the leaves of *R. chrysanthum* (PIABS), the quantum yield of the light energy absorbed by the Photosystem II (PSII) reaction centers for electron transport (φEo), the potential activity of PSII (Fv/Fo) and the captured energy per reaction center of PSII (TRo/RC).

**Figure 1:**
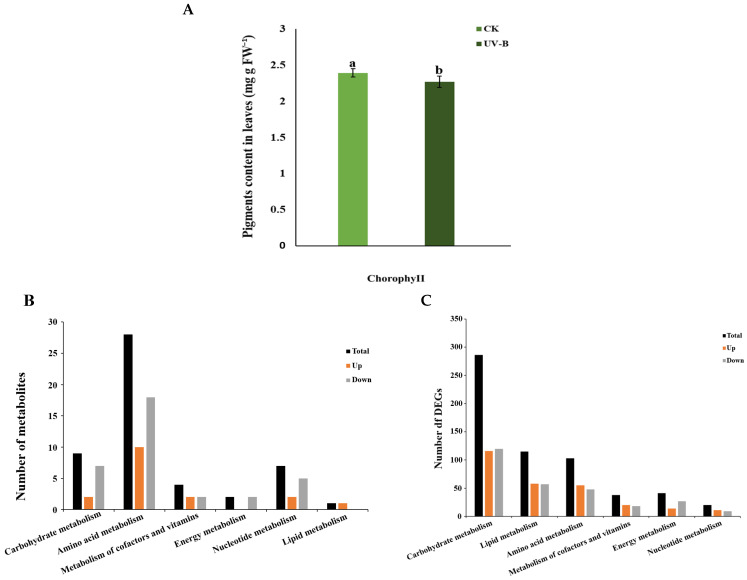
The effects of UV-B radiation on chlorophyll content and metabolic pathways. (**A**) Changes in chlorophyll content of *R. chrysanthum* under UV-B radiation. (**B**) Changes in the relative population of biological macromolecules during the resistance of *R. chrysanthum* to UV-B. “Total amount” refers to the total number of metabolites. (**C**) Statistics of differential genes of the macromolecular metabolic pathway in the process of resistance of *R. chrysanthum* to UV-B. Groups labeled with distinct letters are statistically significant from one another (*p* < 0.05), denoting meaningful differences.

UV-B radiation likewise led to a significant decrease in the chlorophyll content of *R. chrysanthum* (Figure 1A). Taken together, the changes in chlorophyll fluorescence parameters suggest that UV-B radiation can have severe adverse effects on *R. chrysanthum*. Therefore, *R. chrysanthum* will respond to this stress through changes in its own metabolic pathways.

This experiment used LC-MS/MS (liquid chromatography–tandem mass spectrometry) to analyze the metabolism of CK and UV-B-irradiated *R. chrysanthum* leaves (Figure 1B). Many different metabolites were detected in the metabolic pathways of amino acids, lipids, and other macromolecules after UV-B radiation of *R. chrysanthum*. Therefore, we sequenced its leaves. The number of relevant differentially expressed genes in leaves after UV-B radiation was obtained based on sequencing results. The most differential genes (DEGs) were mainly concentrated in the carbohydrate metabolism pathway, with 116 up-regulated genes and 120 down-regulated genes (Figure 1C).

### 2.2. Abscisic Acid (ABA) Production and Signal Transduction in R. chrysanthum in Response to UV-B

The role of the above key metabolic pathways in actively responding to UV-B radiation cannot be ignored, and at the same time, these metabolic pathways are also regulated by phytohormone signaling networks. Therefore, the present study focused on comprehensively analyzing the changes in the levels of various hormones, their biosynthesis, and DEGs in signaling pathways in *R. chrysanthum* under UV-B radiation (Supplementary Tables S1 and S2). This study aimed to present a complete picture of the role of *R. chrysanthum* phytohormones in the defense of *R. chrysanthum* against UV-B radiation.

**Figure 2:**
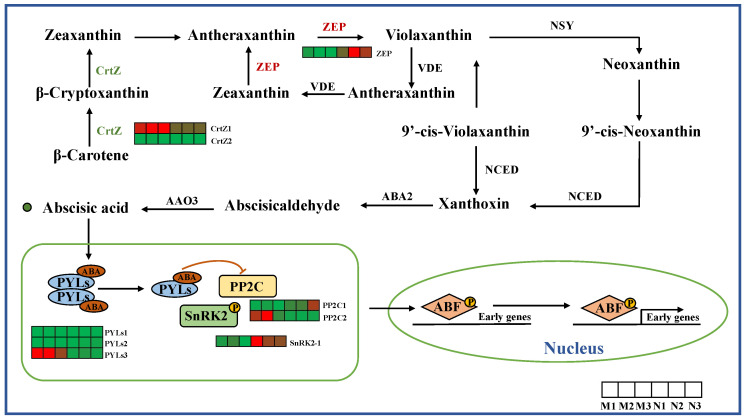
Biosynthesis and signal transduction pathway of ABA. *CrtZ*, beta-carotene 3-hydroxylase; *ZEP*, zeaxanthin epoxidase; *PYL*, pyrooxalate 1-like; *PP2C*, 2C protein phosphatase; *SnRK*, serine/threonine protein kinase. The heat map represents the expression profile of DEGs. Green represents down-regulation and red represents up-regulation. This point indicates the change in compound content in *R. chrysanthum* under UV-B radiation, with red dots being up-regulated and green dots being down-regulated.

The results indicate that during the stage of ABA synthesis, two genes exhibited differential expression. Among them, the expression level of *ZEP* in *R. chrysanthum* irradiated by UV-B was higher, while the expression level of *CrtZ* in control leaves was higher. In the ABA signaling pathway, the expression of *PP2C* and *PYLs* was down-regulated, while the expression of *SnRK2* was up-regulated in *R. chrysanthum* after UV-B radiation (Figure 2).

### 2.3. Gibberellin (GA) Production and Signal Transduction in R. chrysanthum in Response to UV-B

**Figure 3:**
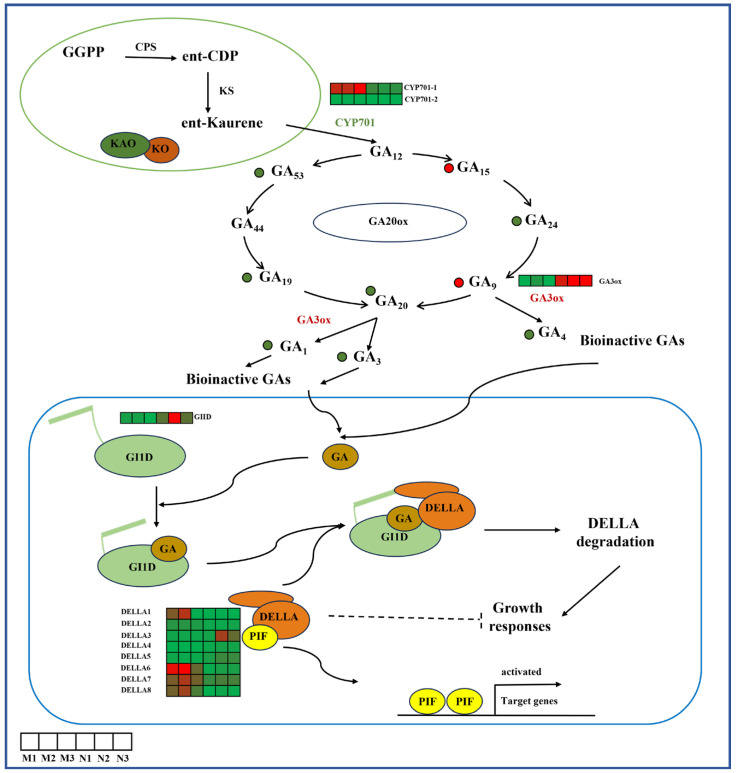
Biosynthetic and signal transduction pathways of GAs. *CYP701*, ent-kaurene oxidase; *GA*, GA; *GA3ox*, GA3-oxidase; *GID1*, GA insensitive dwarf 1; *DELLA*, DELLA protein. The heat map represents the expression profile of DEGs. Green represents down-regulation and red represents up-regulation. This point indicates the change in compound content in *R. chrysanthum* under UV-B radiation, with red dots being up-regulated and green dots being down-regulated.

In GA biosynthesis, after UV-B radiation, the metabolites GA_53_, GA_19_, GA_20_, GA_24_, GA_1_, GA_3_ and GA_4_ were down-regulated, GA_15_ and GA_9_ were up-regulated, *CYP701* expression was down-regulated and *GA3ox* expression was up-regulated. In the GA signal transduction pathway, the expression of *GI1D* was up-regulated and that of *DELLA* was down-regulated after UV-B radiation (Figure 3).

### 2.4. Jasmonic Acid Production and Signal Transduction in R. chrysanthum in Response to UV-B

**Figure 4:**
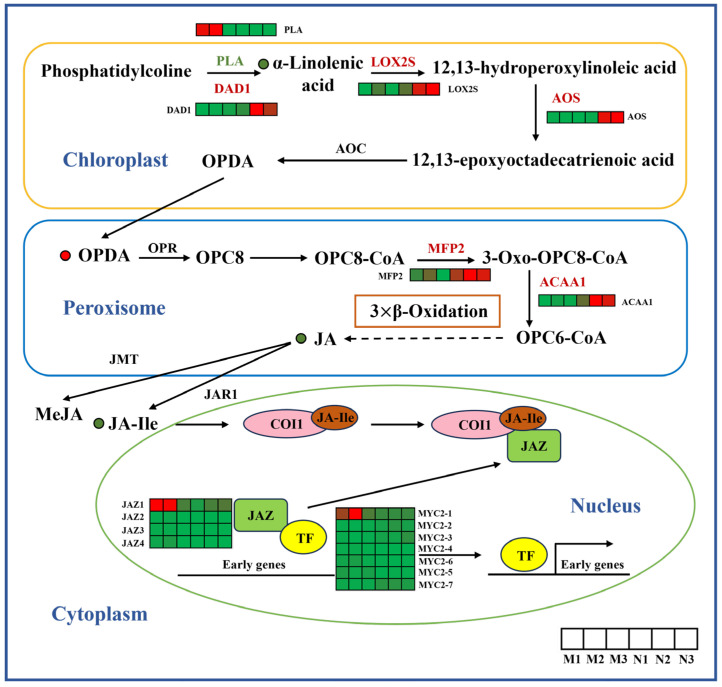
Biosynthetic and signal transduction pathways of JA. *PLA*, phospholipase A2; *DAD1*, phospholipase A1; *LOX2S*, lipoxygenase; *AOS*, allene oxide synthase; *MFP2*, enoyl-CoA hydratase/3-hydroxyacyl-CoA dehydrogenase; *ACAA1*, acetyl-CoA acyltransferase 1; *JAZ*, jasmonate ZIM domain-containing protein. The heat map represents the expression profile of DEGs. Green represents down-regulation and red represents up-regulation. This point indicates the change in compound content in *R. chrysanthum* under UV-B radiation, with red dots being up-regulated and green dots being down-regulated.

This study examined *R. chrysanthum*’s jasmonic acid (JA) biosynthesis and signal transduction pathways to determine the metabolite content and expression patterns of DEGs (Figure 4). Under UV-B radiation, JA-ile, α-linolenic acid and JA contents were higher in the control group (CK group) than in the UV-B group, and OPDA contents were lower than in the UV-B group. Except for *PLA*, *LOX2s*, *DAD1*, *AOS*, *ACAA1* and *MFP25*, biosynthesis genes were up-regulated in leaves after UV-B radiation, which catalyzed a series of reactions in the JA biosynthesis pathway. *MYC2* and *JAZ* expression was down-regulated in response to UV-B radiation, and they are important components of the JA signaling pathway.

### 2.5. Auxin Production and Signal Transduction in R. chrysanthum in Response to UV-B Radiation

**Figure 5:**
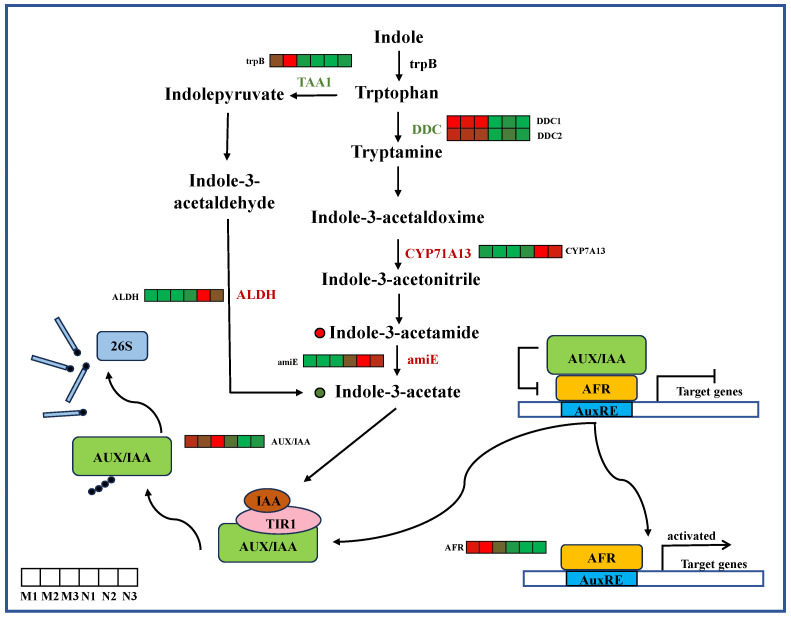
Biosynthetic and signal transduction pathways of auxin. *TAA1*, tryptophan aminotransferase; *CYP71A13*, indoleacetaldoxime dehydratase; ALDH, aldehyde dehydrogenase; *amiE*, amidase; *AUX/IAA*, auxin-responsive protein IAA; *AFR*, auxin response factor. The heat map shows the expression profile of DEGs. Green represents down-regulation and red represents up-regulation. This point indicates the change in compound content in *R. chrysanthum* under UV-B radiation, with red dots being up-regulated and green dots being down-regulated.

The content of Indole-3-acetonitrile was higher and the content of Indole-3-acetate was lower in the leaves of *R. chrysanthum* under UV-B radiation. A total of seven differentially expressed genes of the auxin pathway were obtained, including *TAA1, ALDH*, *TAA1*, *CYP71A13* and *amiE* family genes. After UV-B radiation, the expressions of *DDC* and *TAA1* were down-regulated, while the expressions of *CYP71A13*, *ALDH* and *amiE* were up-regulated. Through differential gene screening, two differentially expressed genes were found in the auxin signaling pathway, namely *AUX/IAA* and *ARF* family genes. Analysis of gene expression profiles of each family showed that *AUX/IAA* and ARF gene families were down-regulated in the leaf midlobes of the UV-B group (Figure 5).

### 2.6. Salicylic Acid (SA) Production and Signal Transduction in R. chrysanthum in Response to UV-B Radiation

**Figure 6:**
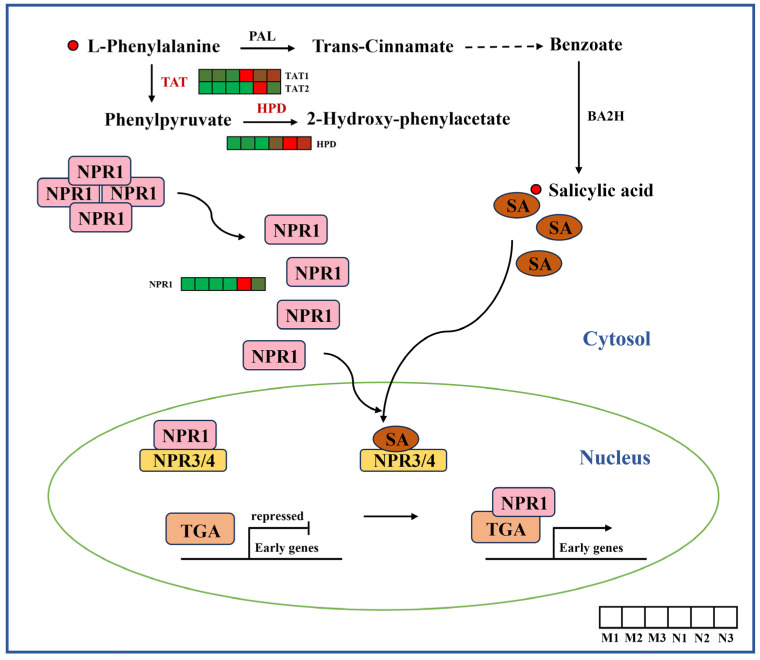
Biosynthetic and signal transduction pathways of SA. *TAT*, tyrosine aminotransferase; *HPD*, 4-hydroxyphenylpyruvate dioxygenase; *NPR1*, nonexpressor of pathogenesis-related genes 1. The heat map shows the expression profile of DEGs. Green represents down-regulation and red represents up-regulation. This point indicates the change in compound content in *R. chrysanthum* under UV-B radiation, with red dots being up-regulated and green dots being down-regulated.

We studied the content of metabolites, the expression of related genes and the SA signal transduction pathway in the process of *R. chrysanthum* SA synthesis. The phenylalanine pathway is the earliest discovered pathway for the synthesis of SA. The results show that UV-B radiation increased the contents of phenylalanine and SA in *R. chrysanthum* leaves, and the expression levels of *TAT* and *HPD* genes in *R. chrysanthum* leaves were higher than those in the CK group. The signal transduction pathway of SA is mainly NPR1-dependent, and the expression level of *NPR1* gene was higher in leaves of the UV-B group (Figure 6).

### 2.7. Cytokinin (CK) Production and Signal Transduction in R. chrysanthum in Response to UV-B Radiation

**Figure 7:**
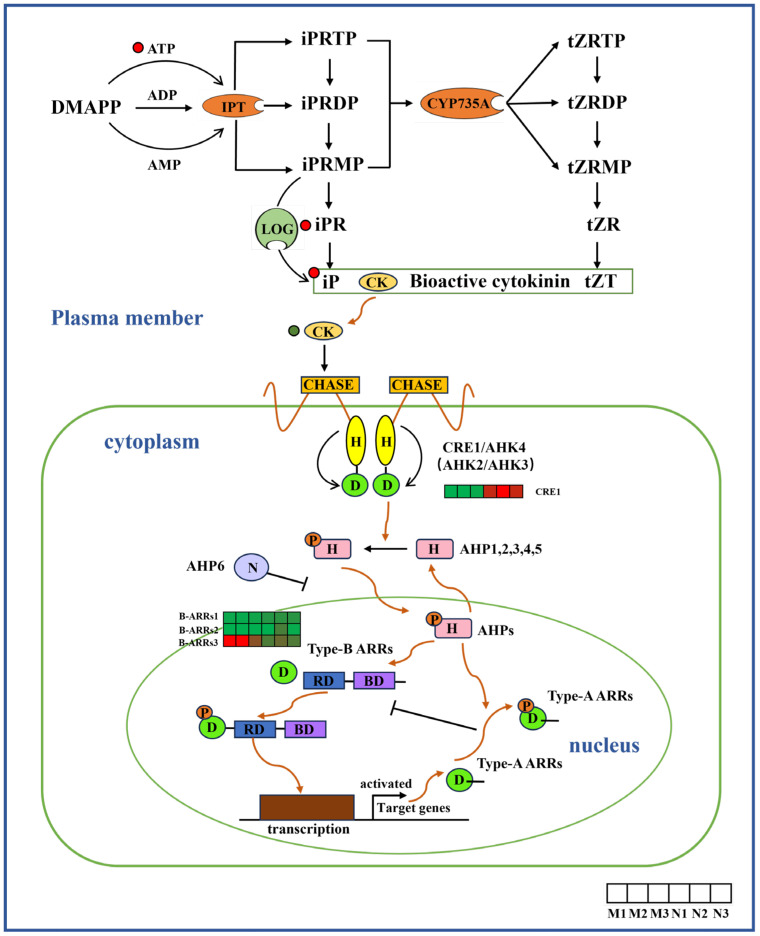
Biosynthetic and signal transduction pathways of CKs. *ATP*, adenosine triphosphate; *CRE1*, CK receptor protein; *Type-B ARRs*, type-B response regulators. The heat map represents the expression profile of DEGs. Green represents down-regulation and red represents up-regulation. This point indicates the change in compound content in *R. chrysanthum* under UV-B radiation, with red dots being up-regulated and green dots being down-regulated.

Through the analysis of the level of CK in the leaves of *R. chrysanthum* before and after UV-B radiation, it was found that the content of other metabolites increased significantly after radiation. In this study, we collected all aspects of genes related to various aspects of CK homeostasis. The results show that the level of *CRE1/AHK4* increased in the CK synthesis pathway due to UV-B radiation in plant leaves, while Type-BARR expression decreased in the CK signal transduction pathway (Figure 7).

### 2.8. Ethylene Production and Signal Transduction in R. chrysanthum in Response to UV-B Radiation

**Figure 8:**
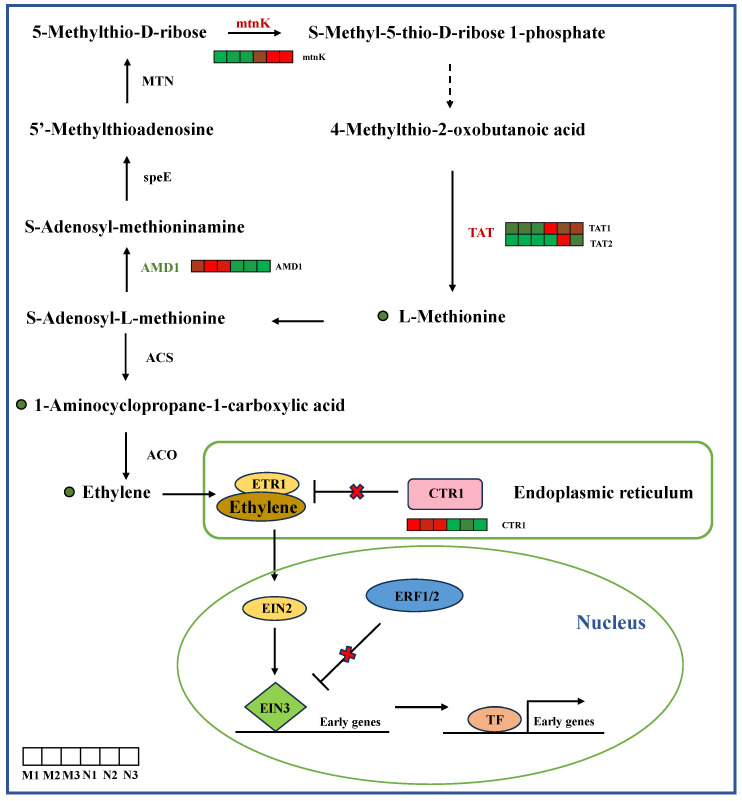
Biosynthetic and signal transduction pathways of ethylene. *mtnK*, 5-methylthioribose kinase; *AMD1*, S-adenosylmethionine decarboxylase; *TAT*, tyrosine aminotransferase. The heat map shows the expression profile of DEGs. Green represents down-regulation and red represents up-regulation. This point indicates the change in compound content in *R. chrysanthum* under UV-B radiation, with red dots being up-regulated and green dots being down-regulated.

It can be seen from Figure 8 that the levels of carboxylic acid and ethylene metabolites in *R. chrysanthum* leaves decreased under UV-B radiation, and the expression levels of JA *mtnK* and *TAT* genes increased in the ethylene biosynthesis pathway, while *AMD1* expression decreased. The expression level of *CTR1* decreased in the ethylene signal transduction pathway.

### 2.9. Correlation Analysis of Various Phytohormones of R. chrysanthum with Photosynthetic Indicators

In order to explore the relationship between various plant hormones under UV-B radiation and the key genes closely associated with them, their regulatory effects on the *R. chrysanthum* photosynthetic system were analyzed. The correlation between DEGs in various plant hormones, their pathways and the above photosynthetic physiological indexes was analyzed.

The result show that the key factors involved in CK regulation of UV-B radiation in *R. chrysanthum* may include *BARR* and *CRE* genes. The *ALDH*, *CYP71A13*, *AUX/IAA* and amiE genes in IAA may be related to protection against UV-B radiation.

Under UV-B radiation, ethylene and SA have two kinds of DEGs with strong correlation, namely *mtnK* and *CTR1*, and *HPD* and *TAT-1*. GA, ABA and JA all involve a DEG in close association, *CYP701-2*, *SNRK2* and *PAL*, respectively.

**Figure 9:**
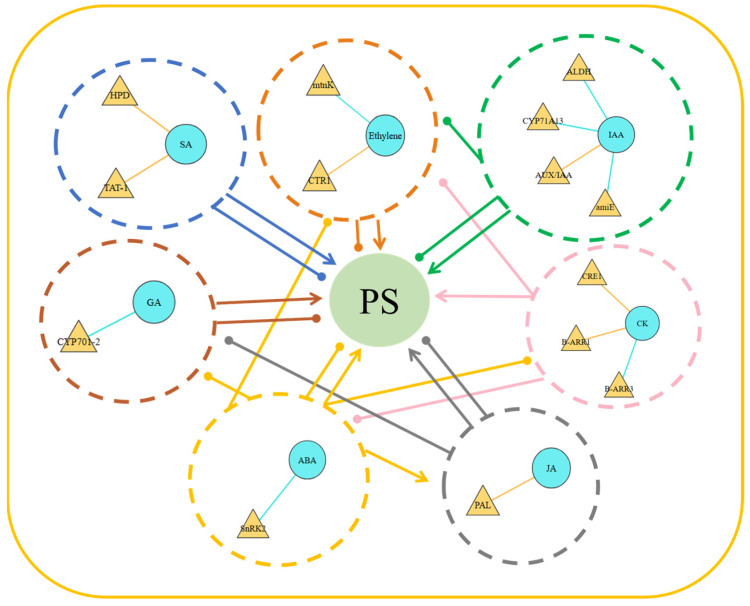
Correlation analysis of phytohormone-related genes and metabolites with photosynthetic indicators. The circles in the dotted circle show hormone-related metabolites, the triangles represent regulatory genes, and the two color lines show two different regulatory relationships between regulatory genes and plant hormone-related metabolites; yellow represents positive regulation and blue represents negative regulation. Colored lines with arrows represent positive regulation, and colored lines with dots represent negative regulation.

This experiment also found that phytohormones have an effect on plant photosynthesis, and that plants reduce photo-oxidative damage under unfavorable conditions through multiple layers of positive and negative regulation. For example, Ck, SA and JAs are promoters of photomorphogenesis, whereas ethylene, ABA, growth factors and GAs are negative regulators (Figure 9).

## 3. Discussion

Light energy induces a series of adversity responses in plants, whereby interactions between phytohormones and ROS can contribute to plant adversity adaptation [28,29]. In plant leaves, chloroplasts can serve as the first line of defense to protect PSII reaction centers from light damage. For this reason, in terms of chlorophyll fluorescence parameters, we found that the fluorescence intensity of *R. chrysanthum* decreased successively after UV-B radiation, indicating that UV-B radiation reduced the reduction ability of the fast reducing PQ library and the slow reducing PQ library, and that the receptor side of the PSII reaction center was damaged. UV-B radiation significantly reduced the maximum photochemical efficiency (Fv/Fm) and potential activity (Fv/Fo) of PSII, whereas the decrease in Tro/Rc reflected an increase in the inactivation of the PSII reaction center [30]. φEo represents the quantum yield of light energy absorbed by the reaction center for electron transfer and is able to reflect the conversion efficiency of light energy [31,32]. UV-B radiation reduced photosynthetic pigments in *R. chrysanthum*, resulting in a decrease in the captured light energy of the photosynthetic system and the absorbed light energy of the photosynthetic reaction center while damaging the photosynthetic system and reducing the effective photosynthetic efficiency. This may be due to the effect of UV-B radiation on the membrane structure, resulting in fewer antenna complexes distributed in the membrane, and thus lower energy conversion and absorption efficiency [33,34]. This down-regulation of the PSII reaction center may be a coping mechanism adopted by *R. chrysanthum* to maintain PSII energy conversion efficiency when they resist UV-B radiation.

Ethylene is the only gas in plant hormones, and it plays an important role in plant growth, development and response to stress. Ethylene synthesis consists of two main enzymatic reactions: (1) 1-aminocyclopropane-1-carboxylicacid (ACC) and methionine react together; (2) ACC is converted to ethylene. These two steps are regulated by ACCsynthase (ACS) and ACCoxidase (ACCoxidase), respectively [35,36,37]. From the perspective of ethylene signaling to the transcriptional regulation of ethylene downstream response factors, a complex ethylene signaling pathway model has been formed. In the correlation analysis, the ethylene content in *R. chrysanthum* was negatively correlated with *mtnk* under UV-B radiation, which may reduce the accumulation of ethylene by increasing gene expression. The *CTR1* gene is positively correlated with the ethylene level in *R. chrysanthum*, and when the ethylene signal is present, ethylene is able to bind to the ethylene receptor (ETR) on the endoplasmic reticulum (ER) membrane and is then transduced by *CTR1* and *EIN2*. This signal is further amplified in an EIN3-mediated transcriptional activation cascade that activates the expression of ethylene response genes [38,39]. In the absence of the ethylene signal, the ethylene receptor may deactivate the kinase activity of *CTR1* in another way, phosphorylating the C-terminal domain of *EIN2*, thereby preventing its participation in ethylene signal transduction [40,41,42]. In conclusion, the accumulation and stabilization of the *EIN3* protein is conducive to the activation of the ethylene signaling pathway, thus enhancing the role of ethylene in plants. The increase in *CTR1* and *EBF1/2* can directly or indirectly inhibit the signal transduction of ethylene and block the transmission of the ethylene signal, which plays an important negative regulatory role. These results suggest that *CTRs* and *mtnK* may be involved in the effect of ethylene on UV-B resistance in *R. chrysanthum* leaves.

ABA is an important plant hormone which has many physiological functions, such as inhibiting growth, promoting shedding, promoting dormancy, causing stomatal closure, regulating seed embryo development, promoting fruit ripening and increasing stress resistance [43,44,45]. Nevertheless, there was no significant change in ABA content after UV-B radiation, which indicates that the leaves of *R. chrysanthum* had certain resistance to UV-B radiation. A number of signaling intermediates associated with ABA responses have been identified by previous studies, and they are tightly controlled by intracellular signal transduction pathways [46]. Progress is currently being made in exploring ABA signaling contributing to the construction of the PYL-PP2C-SnRK2 signaling model. The co-regulatory network of ABA metabolic pathways in *R. chrysanthum* showed that *SnRK2* was negatively correlated with ABA expression levels [23]. According to previous studies, PYR/PYLs are ABA receptors located at the top of the negative regulatory pathway which inhibit PP2Cs from controlling ABA signaling [47]. Therefore, we speculate that *PYL2* may interact with *PP2C* to inhibit phosphatase activity, allow for *SnRK2* activation and target protein phosphorylation, and thus make the leaves have certain resistance to UV-B radiation.

Cytokinin (CK) plays critical regulatory roles in phloem differentiation, chloroplast differentiation, microtubule differentiation, leaf senescence, apical dominance regulation and response to adversity [48]. The decrease in CK levels caused by UV-B radiation may be related to the degree of photosynthesis in plant leaves when they are exposed to a larger photosynthetic capacity per unit leaf area. Studies on Arabidopsis thaliana and tobacco showed that the maximum photosynthetic rate, transpiration rate, stomatal conductance and mass per unit leaf area were reduced when the plants were exposed to light treatment, along with CK [49,50,51]. The results of this experiment show that the CK content in the leaves of *R. chrysanthum* was positively correlated with ARR-B, indicating that UV-B radiation can cause some effects on *R. chrysanthum*. b-type ARRs are a class of transcription factors that activate the transcription of the A-type ARR gene. Previous studies have found that the expression of the a-type ARR gene leads to earlier flowering time, longer root systems, more lateral roots, earlier senescence and reduced CK sensitivity in transgenic plants, and plays a major role among the many transcription factors involved in CK signaling [52]. ARR-B may regulate leaf resistance to radiation through the CK signaling pathway in *R. chrysanthum*.

Auxin is a general term for indole-3-acetic acid (IAA) and its similarly acting analogs that occur naturally in plants [53]. As a signaling compound that promotes and influences plant development and physiological changes, it affects almost the whole process of plant growth and development [54]. UV-B stress changes auxin distribution in plants by affecting the auxin synthesis and transport process and influences downstream genes to make corresponding changes through the auxin signal transduction process. The analysis shows that growth hormone content was negatively regulated by *ALDH*, *CYP71A13* and amiE and positively regulated by *AUX/IAA*. This indicates that UV-B radiation in the growth hormone biosynthesis pathway reduces growth hormone accumulation by increasing gene expression, and that Aux/IAA proteins are transcriptional repressors that mainly inhibit the activity of *ARF* transcription factors [55]. In the signaling pathway, when growth hormone is sensed in the nucleus, it binds to the receptor *TIR1/AFB*, which promotes degradation of the Aux/IAA transcriptional repressor protein, releasing the *ARF* transcription factor and activating downstream gene expression [56].

Salicylic acid (SA), a naturally occurring small-molecule phenolic in the plant kingdom, plays an important role in plant responses to biotic and abiotic stresses as a signaling molecule for plant disease resistance responses. In most plants, SA is synthesized mainly by the phenylalanine pathway. Phenylalanine is catalyzed by the enzyme phenylalanine dehydrogenase to produce cinnamic acid, which is then converted to SA by benzoic acid [57]. The precursor to many polyphenols, such as flavonoids, is cinnamic acid [58]. Studies have shown that UV-B stress can lead to 7–10 times or more up-regulation of endogenous SA content in plants [59,60]. The results of this experiment show that SA content increased in the leaves of *R. chrysanthum* under UV-B radiation. Correlation analysis shows that *TAT1* and *HPD* contents were positively correlated with SA. Therefore, we hypothesized that the higher SA accumulation in the leaves of *R. chrysanthum* under UV-B radiation might be related to the higher expression of the *TAT1* and *HPD* genes. Certainly, this conjecture needs to be verified by further experiments.

Jasmonic acids (JAs) are an important class of lipid-derived phytohormones widely distributed in higher plants and regulate a variety of physiological processes [61]. JAs can act as endogenous signaling molecules to enhance plant resistance to different adversity stresses by regulating gene expression and subsequently accumulating secondary metabolites [62,63]. JA content in the leaves of *R. chrysanthum* was reduced under UV-B radiation, and correlation analysis shows that PLA content was positively correlated with SA. *PLA* is a key enzyme in its metabolic synthesis pathway and is involved in JA biosynthesis. It has been shown that *PLA2* with a molecular mass of 48,000 u was purified from the leaf membrane fractions of faba bean, and it was suggested that it has an important role in the production of linolenic acid [64].

Gibberellins (GAs) are diterpenoid phytohormones that play a crucial role in the whole life cycle of plants [65]. In recent years, numerous studies have shown that GAs significantly promote plant seed germination and seedling growth under adverse environmental conditions, as GAs can alleviate the inhibitory effects of stress on plants; currently there are up to a hundred GAs isolated from various types of organisms named in the order of their discovery [66]. According to our results, the total content of GA compounds was detected to be lower in UV-B-irradiated *R. chrysanthum* leaves versus normal leaves, and correlation analysis shows that the content of the *CYP701* gene in the GA synthesis pathway was negatively correlated with GA. The *CYP701* gene is a related enzyme in the GA biosynthesis pathway. Therefore, the effect of GA on the resistance of *R. chrysanthum* leaves to UV-B radiation needs to be further investigated.

In order to survive, plants must adapt to a wide range of abiotic and biotic stresses. Unfriendly environmental conditions, for example, increased light, a lack of water or low oxygen levels due to abiotic factors such as waterlogging, temperature extremes, salinity and pollutants, may decrease photosynthesis, leading to excess excitation energy in chloroplasts [67,68]. Flexible interactions, complementarities and interactions between phytohormones that regulate photosynthesis are required during plant development and growth to optimize plant dynamics and adaptability in a continuously evolving environment. Chloroplast development is dependent on complex interactions between phytohormones, whereby the plant ensures the minimization of any possible photo-oxidative damage in chloroplasts during light-induced de-yellowing. For example, CKs, JAs and DELLA proteins directly or indirectly promote photomorphogenesis [69,70]. In contrast, ethylene, growth hormone and GAs adversely regulate photomorphogenesis either by pif or by inhibiting B-GATAs [70,71,72]. Plant growth and development are regulated by complementary phytohormones, including photosynthesis between different plant tissues and cells. CK positively regulates photosynthesis by expressing photosynthesis-related genes [72,73]. In contrast, ABA negatively regulates photosynthesis by inhibiting stomatal formation. In addition, interactions between CK and growth hormone regulate plant photosynthesis, whereas cross-talk between growth hormone and ethylene inhibits ethylene biosynthesis and prolongs photosynthesis [74,75].

Plant tolerance to environmental stresses and the manner in which these photoprotective mechanisms are activated vary from species to species, but typically a range of responses occur in plants, some of which are regulated by phytohormones. In fact, studies to more fully understand the effects of hormones on photosynthesis and photoprotection under abiotic stresses are not only important for a better comprehension of the underlying biological processes but may also contribute to the development of new crops with a more robust photosynthetic system.

This study of phytohormonal regulation in *R. chrysanthum* under UV-B radiation provides valuable insights for enhancing plant stress tolerance. These insights can be applied to develop genetically engineered crops with improved UV-B tolerance and higher yields. Additionally, this study highlights the potential for metabolic engineering to boost the production of secondary metabolites with pharmaceutical value. The identified gene expression patterns can also serve as molecular tools for monitoring UV-B stress in plants. Based on these findings, we hypothesized that engineering modifications to these molecular pathways, such as enhancing CK synthesis or SA signaling, may help plants to better adapt and achieve growth advantages under UV-B radiation. This could not only support plant survival in the natural environment but may also provide a theoretical basis for the development of crop varieties tolerant to adversity stress in agricultural production. Overall, the results lay a foundation for developing climate-resilient plants and sustainable agricultural practices.

## 4. Materials and Methods

### 4.1. Plant Materials and Treatment

*R. chrysanthum* was preserved in an artificial climate room at 18 °C (14 h light)/16 °C (10 h dark) under white fluorescent light at 50 umol (photon) m^−2^s^−1^ [18]. The tissue culture seedlings of *R. chrysanthum*, which had the same growth state for 8 months, were selected as the research material.

The experimental materials were divided into experimental (UV-B group) and control groups (CK group). The CK and UV-B groups were transplanted into 1/4 MS medium. The test group was irradiated under UV-B treatment for 2 days, 8 h per day. The control group was irradiated under PAR (photosynthetically active radiation) treatment for two days, eight hours per day [43]. To eliminate inter-individual differences, three biological replicates were performed in this study. The PAR irradiation treatment involved the placement of a 400 nm photofilm (Edmund, Filter Long 2IN SQ, Barrington, NJ, USA). The UV-B radiation treatment involved the measurement of filters (Edmund, Filter Long 2IN SQ, Barrington, NJ, USA). The artificial UV-B radiation source was a UV-B TL 20W/01RS fluorescent tube (Philips, UltravioletB TL 20 W/01 RS, Amsterdam, The Netherlands). The effective received irradiance of the samples was UV-B: 2.3 W/m2, PAR: 50 umol/ (m^2^·s).

### 4.2. Identification and Quantification of Metabolites

We strictly followed the experimental steps of a previous study [26]. Using vacuum freeze-drying technology, we placed the biological samples in a lyophilizer (Scientz-100F, Ningbo Scientz Biotechnology Co., Ltd., Ningbo, China), then ground (30 Hz, 1.5 min) the samples to a powder form by using a grinder (MM400, Retsch, Haan, Germany). Next, we weighed 50 mg of sample powder using an electronic balance (MS105DΜ) and added 1200 μL of −20 °C pre-cooled 70% methanolic aqueous internal standard extract (less than 50 mg added at the rate of 1200 μL extractant per 50 mg sample) [43]. Vortexing was performed every 30 min for 30 s for a total of 6 times. After centrifugation (12,000 rpm, 3 min), the supernatant was withdrawn, and the sample was filtered with a microporous membrane with a pore size of 0.22 μm and stored in an injection vial for LC-MS/MS (liquid chromatography–tandem mass spectrometry) analysis. Analysis of sample extracts was performed using an LC-MS/MS system and a tandem mass spectrometer. Metabolite profiling data were obtained from different samples, and the chromatographic peaks of all substances were integrated and corrected. Metabolites with FC (fold change) ≥ 2 and FC ≤ 0.5 were selected as differential metabolites.

### 4.3. cDNA Library Construction and Transcriptomics Data Analysis

Transcriptomics experiments were conducted strictly according to the previous experimental instructions [76].

The total RNA was extracted from the samples using the CTAB method. The total RNA samples were treated by using an rRNA removal method, and the rRNA was hybridized with a DNA probe. The hybrid DNA/RNA chain was digested by RNaseH, the DNA probe was digested by DNaseI and the RNA was purified. The resulting RNA was fragmented by using a disruption buffer, reverse-transcribed with randomized N6 primers, and then synthesized into cDNA duplexes, forming synthetic double-stranded DNA that was sprawlingly phosphorylated at the 5′ end, formed a sticky end with a prominent “a” at the 3′ end, and attached to a bulge-like junction with a prominent “T” at the 3′ end. The ligated product was amplified by PCR with specific primers; the PCR product was heat-denatured to a single strand, and then the single-stranded DNA was cyclized with a bridge primer to obtain a single-stranded circular DNA library, which was finally sequenced on the machine [47].

The transcriptome sequencing in this study was performed using the MGISEQ-2000 platform of BGI Genomics Co., Ltd. (Shenzhen, China) and a total of 58 Gb was sequenced, and 93,034 unigenes were obtained after assembly and de-redundancy.

To ensure the reliability of the results, we relied on the filtering software SOAPnuke (v1.4.0) [77] and used Bowtie2 (v2.2.5) [78] to compare the clean reads to the reference gene sequences to obtain the comparison results. The gene and transcript expression levels were estimated using RSEM (v 1.2.8) [79]. To identify differentially expressed genes following experimental treatments, the expression levels of each transcript were measured using the FPKM (Fragments Per Kilobase of transcript per Million mapped reads) method. Subsequently, the DESeq2 method, based on the negative binomial distribution, was employed to analyze differentially expressed genes in response to the experimental treatments. Genes were identified as differentially expressed when they exhibited an FC (fold change) greater than 1 and q-value (adjusted *p*-value) < 0.05.

Functional annotation and classification of Unigenes were conducted using publicly available database resources, including KEGG, Pfam and SwissProt. The annotation process involved sequence alignment of Unigene datasets against the reference sequences in these databases using BLAST-based comparison algorithms.

### 4.4. Chlorophyll Fluorescence Measurements

The CK and UV-B groups were dark-treated for 20 min before measurement, and finally, chlorophyll fluorescence parameters were obtained using IMAGING PAM m-series (Walz, Effeltrich, Germany) [80].

### 4.5. Rapid Fluorescence Detection

Our method was in compliance with previous experimental descriptions [81]. In this experiment, the operation steps of the Handy-PEA instrument were as follows: We clamped the leaf clip on the front of the leaf and allowed for dark adaptation for 20 min. In an attempt to minimize the effect of leaf heterogeneity on the detection results, eight detection points were selected for each leaf: four detection points were selected for each leaf along both sides of the main leaf veins, and four detection points were equally spaced from the leaf tip to the leaf base.

After dark adaptation, we connected the instrument probe to the blade clamp on the blade and opened the slide switch to expose the measuring hole to the laser light source.

According to the pre-set LED light source of 3000 μmol m^−2^s^−1^, the detection time was 1s for fast fluorescence signal acquisition. Finally, the fluorescence signals of 8 detection points on each leaf were averaged and used as the final fast fluorescence data of the sample.

### 4.6. Determination of Chlorophyll Content

A total of 1.0 g of leaves of *R. chrysanthum* from the CK and UV-B groups was weighed, ground repeatedly in 80% acetone solution and filtered, and the filtrate was collected. The absorbance of the extracts at 663 nm and 645 nm was determined by using a colorimetric method. This process was repeated three times. The mass concentrations of chlorophyll a and chlorophyll b in the extracts were calculated according to the formula below, and then the chlorophyll content in the leaves was calculated and expressed as the mass of chlorophyll contained in each gram of fresh weight of leaf tissue (mg/g). The formula was calculated as follows, where ρ is the mass concentration of chlorophyll mg/L, calculated by the formula below; V is the total volume of the sample extract in mL; and m is the sample mass.

$$\rho_{a}=12.72A_{663}-2.59A_{645}$$

$$\rho_{b}=22.88A_{645}-4.67A_{663}$$

$$\rho_{chlorophyll\;content}=\rho_{a}+\rho_{b}$$

$$chlorophyll\;content=\rho *V/m*1000\left(mg/g\right)$$

### 4.7. Statistical Analysis

The trials were executed on three occasions, employing a fully randomized design, and the data were analyzed utilizing IBM SPSS Statistics version 26. A one-way ANOVA was utilized to evaluate the statistical significance of the findings. Upon identifying significant disparities, Duncan’s multiple range test was implemented to pinpoint the specific mean differences at a significance threshold of *p* < 0.05.

Genomic expression data were processed through the interactive data mining system named Dr.Tom [82]. The relationships between plant hormones and differentially expressed genes (DEGs) derived from RNA sequencing were examined using Pearson’s correlation coefficient, with a stringent correlation threshold set at 0.9 and a significance level of *p* < 0.05.

The dataset obtained from the above experiments was previously used to draw conclusions on other issues regarding the response of *R. chrysathum* to UV-B [27,83,84].

## 5. Conclusions

**Figure 10:**
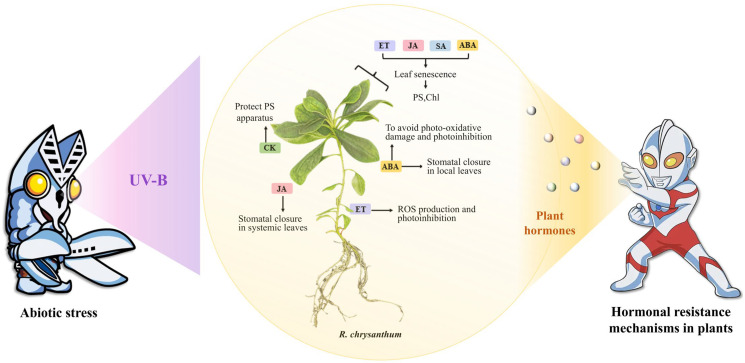
Plant hormone control network in *R. chrysanthum* under UV-B irradiation.

The present study provides a comprehensive analysis of phytohormonal regulation in *R. chrysanthum* under UV-B radiation, revealing the complex interplay between hormonal signaling and metabolic responses in plant stress tolerance. Our findings indicate that UV-B radiation significantly impacts the photosynthetic capacity of *R. chrysanthum*, leading to a decline in photosynthesis efficiency and damage to the photosynthetic system. This is accompanied by alterations in the levels of various phytohormones, their biosynthesis, and the expression of related signaling genes, which collectively contribute to the plant’s defense mechanisms against UV-B stress (Figure 10).

Additionally, while UV-B radiation primarily imposes stress on the plant, our study also hints at the potential for UV-B exposure to induce adaptive responses that may enhance certain growth-related processes in *R. chrysanthum*. This aligns with the concept of radiation hormesis, where radiation can stimulate plant growth through the activation of specific hormonal pathways and defense mechanisms. These findings not only expand our understanding of hormonal regulatory mechanisms in plant resistance to UV-B radiation but also lay the groundwork for developing strategies to enhance plant tolerance to adverse environmental stresses.

## Data Availability

The data used in this study are available from the corresponding author on submission of a reasonable request.

## Supplementary Materials

The following supporting information can be downloaded at https://www.mdpi.com/article/10.3390/ijms26041545/s1. References [81,85] are cited in the Supplementary Materials.

## Abbreviations

UV-B	Ultraviolet-B radiation
*R. chrysanthum*	*Rhododendron chrysanthum* Pall.
LC-MS/MS	Liquid chromatography–tandem mass spectrometry
DEGs	Differentially expressed genes
ABA	Abscisic acid
GA	Gibberellin
JA	Jasmonic acid
IAA	Indole-3-acetic acid
SA	Salicylic acid
CK	Cytokinin
CK group	Control group
PAR	Photosynthetically active radiation
FPKM	Fragments Per Kilobase of transcript per Million mapped reads
KEGG	Kyoto Encyclopedia of Genes and Genomes
Pfam	Protein family
SwissProt	Swiss Protein
ANOVA	Analysis of variance
SPSS	Statistical Package for the Social Sciences
PSII	Photosystem II

## References

[1] Chen Z., Dong Y., Huang X. (2022). Plant responses to UV-B radiation: Signaling, acclimation and stress tolerance. Stress. Biol..

[2] Demarsy E., Goldschmidt-Clermont M., Ulm R. (2018). Coping with ’Dark Sides of the Sun’ through Photoreceptor Signaling. Trends Plant Sci..

[3] Kami C., Lorrain S., Hornitschek P., Fankhauser C. (2010). Light-regulated plant growth and development. Curr. Top. Dev. Biol..

[4] Rizzini L., Favory J.J., Cloix C., Faggionato D., O’Hara A., Kaiserli E., Baumeister R., Schäfer E., Nagy F., Jenkins G.I. (2011). Perception of UV-B by the Arabidopsis UVR8 protein. Science.

[5] Roy S. (2017). Impact of UV Radiation on Genome Stability and Human Health. Adv. Exp. Med. Biol..

[6] Ven Vanitha Jenishree S., Doss A., Kabir N.A. (2024). The impact of supplemental UV-B radiation on growth and biochemical constituents in *Vigna unguiculata* L. Walp and *Pisum sativum* L. Sustain. Chem. One World.

[7] Parihar P., Singh S., Singh R., Singh V.P., Prasad S.M. (2015). Changing scenario in plant UV-B research:UV-B from a generic stressor to a specific regulator. J. Photochem. Photobiol. B Biol..

[8] Falara V., Amarasinghe R., Poldy J., Pichersky E., Barrow R.A., Peakall R. (2013). The production of a key floral volatile is dependent on UV light in a sexually deceptive orchid. Ann. Bot..

[9] Chen Y., Wang Y., Liang X., Zhang Y., Fernie A.R. (2023). Mass spectrometric exploration of phytohormone profiles and signaling networks. Trends Plant Sci..

[10] Das S., Shil S., Rime J., Alice A.K., Yumkhaibam T., Mounika V., Singh A.P., Kundu M., Lalhmangaihzuali H.P., Hazarika T.K. (2025). Phytohormonal signaling in plant resilience: Advances and strategies for enhancing abiotic stress tolerance. Plant Growth Regul..

[11] Shi Y., Yang S., Zhang D.-P. (2014). ABA Regulation of the Cold Stress Response in Plants. Abscisic Acid: Metabolism, Transport and Signaling.

[12] Guo Y., Yan J., Su Z., Chang J., Yang J., Wei C., Zhang Y., Ma J., Zhang X., Li H. (2021). Abscisic Acid Mediates Grafting-Induced Cold Tolerance of Watermelon via Interaction With Melatonin and Methyl Jasmonate. Front. Plant Sci..

[13] Wang Y., Mostafa S., Zeng W., Jin B. (2021). Function and Mechanism of Jasmonic Acid in Plant Responses to Abiotic and Biotic Stresses. Int. J. Mol. Sci..

[14] Singh V.P., Kumar J., Singh S., Prasad S.M. (2014). Dimethoate modifies enhanced UV-B effects on growth, photosynthesis and oxidative stress in mung bean (*Vigna radiata* L.) seedlings: Implication of salicylic acid. Pestic. Biochem. Physiol..

[15] Shi Y., Tian S., Hou L., Huang X., Zhang X., Guo H., Yang S. (2012). Ethylene signaling negatively regulates freezing tolerance by repressing expression of CBF and type-A ARR genes in Arabidopsis. Plant Cell.

[16] Lantzouni O., Alkofer A., Falter-Braun P., Schwechheimer C. (2020). Growth-Regulating FactorS Interact with DELLAs and Regulate Growth in Cold Stress. Plant Cell.

[17] Zhang Q., Li Y., Sun L., Chu S., Xu H., Zhou X. (2023). Integration of transcriptomic and proteomic analyses of Rhododendron chrysanthum Pall. in response to cold stress in the Changbai Mountains. Mol. Biol. Rep..

[18] Liu M., Sun Q., Cao K., Xu H., Zhou X. (2023). Acetylated Proteomics of UV-B Stress-Responsive in Photosystem II of Rhododendron chrysanthum. Cells.

[19] Yu W., Gong F., Cao K., Zhou X., Xu H. (2024). Multi-Omics Analysis Reveals the Molecular Mechanisms of the Glycolysis and TCA Cycle Pathways in Rhododendron chrysanthum Pall. under UV-B Stress. Agronomy.

[20] Yu W., Gong F., Xu H., Zhou X. (2024). Molecular Mechanism of Exogenous ABA to Enhance UV-B Resistance in Rhododendron chrysanthum Pall. by Modulating Flavonoid Accumulation. Int. J. Mol. Sci..

[21] Sulpice R., Pyl E.T., Ishihara H., Trenkamp S., Steinfath M., Witucka-Wall H., Gibon Y., Usadel B., Poree F., Piques M.C. (2009). Starch as a major integrator in the regulation of plant growth. Proc. Natl. Acad. Sci. USA.

[22] Fukushima A., Kusano M., Redestig H., Arita M., Saito K. (2009). Integrated omics approaches in plant systems biology. Curr. Opin. Chem. Biol..

[23] Zhu C., Xiaoyu L., Junlan G., Yun X., Jie R. (2020). Integrating transcriptomic and metabolomic analysis of hormone pathways in Acer rubrum during developmental leaf senescence. BMC Plant Biol..

[24] Jin J., Zhang H., Zhang J., Liu P., Chen X., Li Z., Xu Y., Lu P., Cao P. (2017). Integrated transcriptomics and metabolomics analysis to characterize cold stress responses in Nicotiana tabacum. BMC Genom..

[25] Fasano C., Diretto G., Aversano R., D’Agostino N., Di Matteo A., Frusciante L., Giuliano G., Carputo D. (2016). Transcriptome and metabolome of synthetic Solanum autotetraploids reveal key genomic stress events following polyploidization. New Phytol..

[26] Sun Q., Li X., Sun L., Sun M., Xu H., Zhou X. (2024). Plant hormones and phenolic acids response to UV-B stress in Rhododendron chrysanthum pall. Biol. Direct.

[27] Yu W., Zhou X., Meng J., Zhou X., Xu H. (2025). Multi-Omics Research Reveals the Effects of the ABA-Regulated Phenylpropanoid Biosynthesis Pathway on the UV-B Response in Rhododendron chrysanthum Pall. Plants.

[28] Müller M., Munné-Bosch S. (2021). Hormonal impact on photosynthesis and photoprotection in plants. Plant Physiol..

[29] Razzaq K., Du J. (2024). Phytohormonal Regulation of Plant Development in Response to Fluctuating Light Conditions. J. Plant Growth Regul..

[30] Strasser B.J., Strasser R.J. (1995). Measuring fast fluorescence transients to address environmental questions: The JIP-test. Photosynthesis: From Light to Biosphere.

[31] Bhowmick A., Hussein R., Bogacz I., Simon P.S., Ibrahim M., Chatterjee R., Doyle M.D., Cheah M.H., Fransson T., Chernev P. (2023). Structural evidence for intermediates during O2 formation in photosystem II. Nature.

[32] Li H., Nakajima Y., Nango E., Owada S., Yamada D., Hashimoto K., Luo F., Tanaka R., Akita F., Kato K. (2024). Oxygen-evolving photosystem II structures during S1–S2–S3 transitions. Nature.

[33] Zhao L.S., Li K., Wang Q.M., Song X.Y., Su H.N., Xie B.B., Zhang X.Y., Huang F., Chen X.L., Zhou B.C. (2017). Nitrogen Starvation Impacts the Photosynthetic Performance of Porphyridium cruentum as Revealed by Chlorophyll a Fluorescence. Sci. Rep..

[34] Kalaji H.M., Bąba W., Gediga K., Goltsev V., Samborska I.A., Cetner M.D., Dimitrova S., Piszcz U., Bielecki K., Karmowska K. (2018). Chlorophyll fluorescence as a tool for nutrient status identification in rapeseed plants. Photosynth. Res..

[35] Houben M., Van de Poel B. (2019). 1-Aminocyclopropane-1-Carboxylic Acid Oxidase (ACO): The Enzyme That Makes the Plant Hormone Ethylene. Front. Plant Sci..

[36] Pattyn J., Vaughan-Hirsch J., Van de Poel B. (2021). The regulation of ethylene biosynthesis: A complex multilevel control circuitry. New Phytol..

[37] Van de Poel B., Van Der Straeten D. (2014). 1-aminocyclopropane-1-carboxylic acid (ACC) in plants: More than just the precursor of ethylene!. Front. Plant Sci..

[38] Zhou Y., Ma B., Tao J.J., Yin C.C., Hu Y., Huang Y.H., Wei W., Xin P.Y., Chu J.F., Zhang W.K. (2022). Rice EIL1 interacts with OsIAAs to regulate auxin biosynthesis mediated by the tryptophan aminotransferase MHZ10/OsTAR2 during root ethylene responses. Plant Cell.

[39] Binder B.M. (2020). Ethylene signaling in plants. J. Biol. Chem..

[40] Ju C., Yoon G.M., Shemansky J.M., Lin D.Y., Ying Z.I., Chang J., Garrett W.M., Kessenbrock M., Groth G., Tucker M.L. (2012). CTR1 phosphorylates the central regulator EIN2 to control ethylene hormone signaling from the ER membrane to the nucleus in Arabidopsis. Proc. Natl. Acad. Sci. USA.

[41] Wang Q. (2024). Study on the expression regulation of the CTR1 gene in the ethylene signaling pathway. Biochem. Biophys. Res. Commun..

[42] Wang X., Wen H., Suprun A., Zhu H. (2025). Ethylene Signaling in Regulating Plant Growth, Development, and Stress Responses. Plants.

[43] Sun Q., Zhou X., Yang L., Xu H., Zhou X. (2023). Integration of Phosphoproteomics and Transcriptome Studies Reveals ABA Signaling Pathways Regulate UV-B Tolerance in Rhododendron chrysanthum Leaves. Genes.

[44] Jiang Y., Jiang S., Liu L. (2025). Understanding the multifaceted role of ABA signaling in orchestrating plant developmental transition. Stress. Biol..

[45] Li C., Pan Y., Cui J., Lu X., Yu W. (2025). Mechanism of ABA in Plants Exposed to Cold Stress. Agronomy.

[46] Nakashima K., Yamaguchi-Shinozaki K. (2013). ABA signaling in stress-response and seed development. Plant Cell Rep..

[47] Zhang Q., Li Y., Cao K., Xu H., Zhou X. (2023). Transcriptome and proteome depth analysis indicate ABA, MAPK cascade and Ca(2+) signaling co-regulate cold tolerance in Rhododendron chrysanthum Pall. Front. Plant Sci..

[48] Kieber J.J., Schaller G.E. (2018). Cytokinin signaling in plant development. Development.

[49] Boonman A., Prinsen E., Gilmer F., Schurr U., Peeters A.J., Voesenek L.A., Pons T.L. (2007). Cytokinin import rate as a signal for photosynthetic acclimation to canopy light gradients. Plant Physiol..

[50] Teramura A.H., Sullivan J.H. (1994). Effects of UV-B radiation on photosynthesis and growth of terrestrial plants. Photosynth. Res..

[51] Mmbando G.S., Ngongolo K. (2024). Environmental & health impacts of ultraviolet radiation: Current trends and mitigation strategies. Discov. Sustain..

[52] Argueso C.T., Raines T., Kieber J.J. (2010). Cytokinin signaling and transcriptional networks. Curr. Opin. Plant Biol..

[53] Casanova-Sáez R., Mateo-Bonmatí E., Ljung K. (2021). Auxin Metabolism in Plants. Cold Spring Harb. Perspect. Biol..

[54] Leyser O. (2018). Auxin Signaling. Plant Physiol..

[55] Ulmasov T., Murfett J., Hagen G., Guilfoyle T.J. (1997). Aux/IAA proteins repress expression of reporter genes containing natural and highly active synthetic auxin response elements. Plant Cell.

[56] Benjamins R., Scheres B. (2008). Auxin: The looping star in plant development. Annu. Rev. Plant Biol..

[57] Lee H.I., León J., Raskin I. (1995). Biosynthesis and metabolism of salicylic acid. Proc. Natl. Acad. Sci. USA.

[58] Rohde A., Morreel K., Ralph J., Goeminne G., Hostyn V., De Rycke R., Kushnir S., Van Doorsselaere J., Joseleau J.P., Vuylsteke M. (2004). Molecular phenotyping of the pal1 and pal2 mutants of Arabidopsis thaliana reveals far-reaching consequences on phenylpropanoid, amino acid, and carbohydrate metabolism. Plant Cell.

[59] Catinot J., Buchala A., Abou-Mansour E., Métraux J.P. (2008). Salicylic acid production in response to biotic and abiotic stress depends on isochorismate in Nicotiana benthamiana. FEBS Lett..

[60] Wildermuth M.C., Dewdney J., Wu G., Ausubel F.M. (2001). Isochorismate synthase is required to synthesize salicylic acid for plant defence. Nature.

[61] Delfin J.C., Kanno Y., Seo M., Kitaoka N., Matsuura H., Tohge T., Shimizu T. (2022). AtGH3.10 is another jasmonic acid-amido synthetase in Arabidopsis thaliana. Plant J. Cell Mol. Biol..

[62] Marquis V., Smirnova E., Graindorge S., Delcros P., Villette C., Zumsteg J., Heintz D., Heitz T. (2022). Broad-spectrum stress tolerance conferred by suppressing jasmonate signaling attenuation in Arabidopsis JASMONIC ACID OXIDASE mutants. Plant J. Cell Mol. Biol..

[63] Yang S., Cao Q., Peng K., Xie J. (2022). Jasmonic Acid-Treated Cotton Plant Leaves Impair Larvae Growth Performance, Activities of Detoxification Enzymes, and Insect Humoral Immunity of Cotton Bollworm. Neotrop. Entomol..

[64] Jung K.M., Kim D.K. (2000). Purification and characterization of a membrane-associated 48-kilodalton phospholipase A(2) in leaves of broad bean. Plant Physiol..

[65] Davière J.M., Achard P. (2013). Gibberellin signaling in plants. Development.

[66] Liu J., Wu Y., Dong G., Zhu G., Zhou G. (2023). Progress of Research on the Physiology and Molecular Regulation of Sorghum Growth under Salt Stress by Gibberellin. Int. J. Mol. Sci..

[67] Asada K. (2006). Production and scavenging of reactive oxygen species in chloroplasts and their functions. Plant Physiol..

[68] Asada K. (1999). THE WATER-WATER CYCLE IN CHLOROPLASTS: Scavenging of Active Oxygens and Dissipation of Excess Photons. Annu. Rev. Plant Physiol. Plant Mol. Biol..

[69] Tsuchiya Y., Vidaurre D., Toh S., Hanada A., Nambara E., Kamiya Y., Yamaguchi S., McCourt P. (2010). A small-molecule screen identifies new functions for the plant hormone strigolactone. Nat. Chem. Biol..

[70] Yang D.L., Yao J., Mei C.S., Tong X.H., Zeng L.J., Li Q., Xiao L.T., Sun T.P., Li J., Deng X.W. (2012). Plant hormone jasmonate prioritizes defense over growth by interfering with gibberellin signaling cascade. Proc. Natl. Acad. Sci. USA.

[71] Lau O.S., Deng X.W. (2012). The photomorphogenic repressors COP1 and DET1: 20 years later. Trends Plant Sci..

[72] Mayzlish-Gati E., LekKala S.P., Resnick N., Wininger S., Bhattacharya C., Lemcoff J.H., Kapulnik Y., Koltai H. (2010). Strigolactones are positive regulators of light-harvesting genes in tomato. J. Exp. Bot..

[73] Brenner W.G., Schmülling T. (2012). Transcript profiling of cytokinin action in Arabidopsis roots and shoots discovers largely similar but also organ-specific responses. BMC Plant Biol..

[74] Bianchetti R.E., Cruz A.B., Oliveira B.S., Demarco D., Purgatto E., Peres L.E.P., Rossi M., Freschi L. (2017). Phytochromobilin deficiency impairs sugar metabolism through the regulation of cytokinin and auxin signaling in tomato fruits. Sci. Rep..

[75] Yuan Y., Xu X., Gong Z., Tang Y., Wu M., Yan F., Zhang X., Zhang Q., Yang F., Hu X. (2019). Auxin response factor 6A regulates photosynthesis, sugar accumulation, and fruit development in tomato. Hortic. Res..

[76] Yu W., Gong F., Zhou X., Xu H., Lyu J., Zhou X. (2024). Comparative Metabolomics and Transcriptome Studies of Two Forms of Rhododendron chrysanthum Pall. under UV-B Stress. Biology.

[77] Chen Y., Chen Y., Shi C., Huang Z., Zhang Y., Li S., Li Y., Ye J., Yu C., Li Z. (2018). SOAPnuke: A MapReduce acceleration-supported software for integrated quality control and preprocessing of high-throughput sequencing data. Gigascience.

[78] Langmead B., Salzberg S.L. (2012). Fast gapped-read alignment with Bowtie 2. Nat. Methods.

[79] Li B., Dewey C.N. (2011). RSEM: Accurate transcript quantification from RNA-Seq data with or without a reference genome. BMC Bioinform..

[80] Zhou X., Lyu J., Sun L., Dong J., Xu H. (2021). Metabolic programming of Rhododendron chrysanthum leaves following exposure to UVB irradiation. Funct. Plant Biol..

[81] Zhou X., Yu W., Gong F., Xu H., Lyu J., Zhou X. (2024). Golden 2-like Transcription Factors Regulate Photosynthesis under UV-B Stress by Regulating the Calvin Cycle. Plants.

[82] Zhang A., Fang J., Wang J., Xie X., Chen H.-J., He G. (2022). Interrogation on the Cellular Nano-Interface and Biosafety of Repeated Nano-Electroporation by Nanostraw System. Biosensors.

[83] Liu M., Gong F., Yu W., Cao K., Xu H., Zhou X. (2024). The Rhododendron Chrysanthum Pall.s’ Acetylation Modification of Rubisco Enzymes Controls Carbon Cycling to Withstand UV-B Stress. Biomolecules.

[84] Gong F., Meng J., Xu H., Zhou X. (2024). The Molecular Mechanism Regulating Flavonoid Production in Rhododendron chrysanthum Pall. Against UV-B Damage Is Mediated by RcTRP5. Int. J. Mol. Sci..

[85] Gong F., Zhou X., Yu W., Xu H., Zhou X. (2024). Carotenoid Accumulation in the Rhododendron chrysanthum Is Mediated by Abscisic Acid Production Driven by UV-B Stress. Plants.

