# Supplementary material for: Integrating Transcriptomics and Metabolomics to Comprehensively Analyze Phytohormone Regulatory Mechanisms in Rhododendron chrysanthum Pall. Under UV-B Radiation
Source: Int J Mol Sci. 2025 Feb 12;26(4):1545. doi: 10.3390/ijms26041545 (PMC11855671; doi:10.3390/ijms26041545)
Supplement: Supplementary file 1 [file ijms-26-01545-s001.zip › Supplementary Figures.pdf]

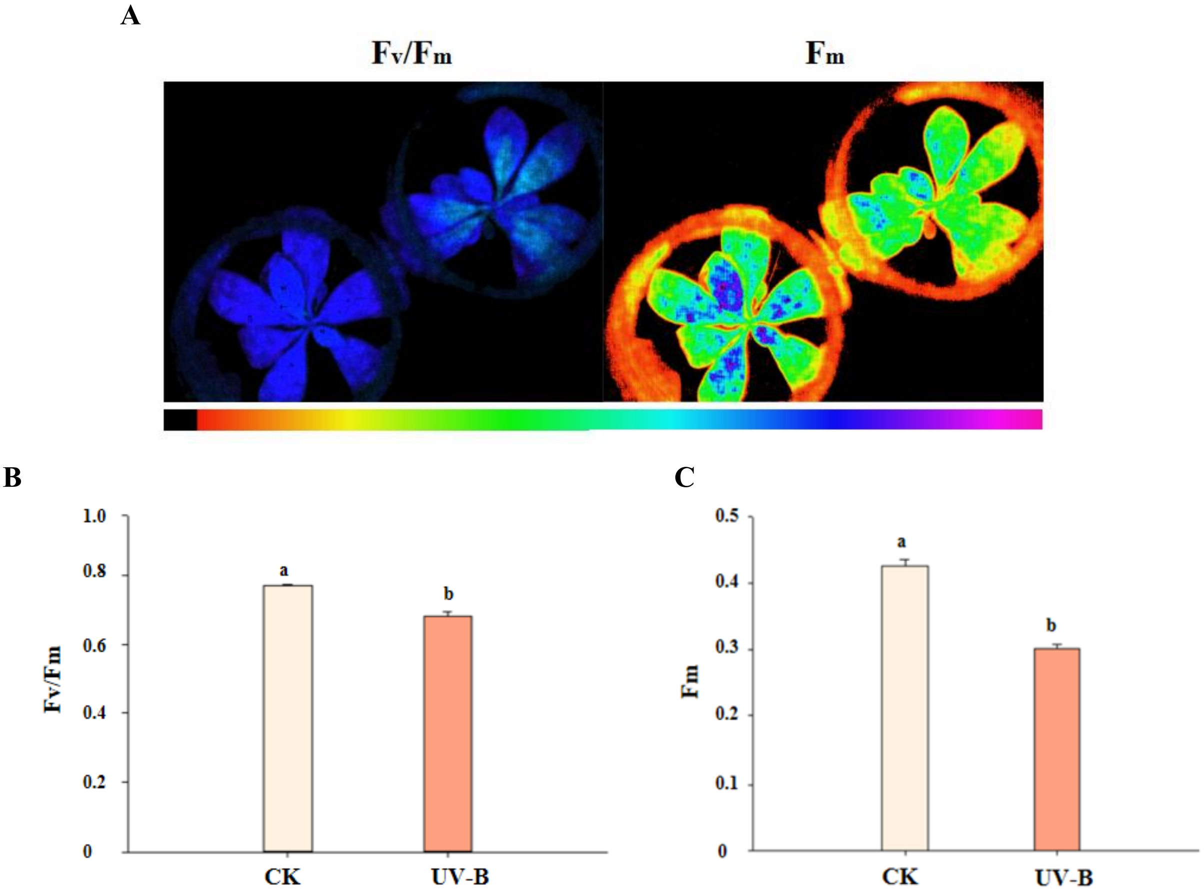

Supplementary Figure S1. Photochemical activity analysis of *R. chrysanthum* under UV-B radiation. (A) chlorophyll fluorescence imaging (note: left control, right UV-B radiation); (B) The bar graph of Fm; (C) The bar graph of Fv/Fm. The bar chart displays the average heights for each group, derived from three biological replicates (n = 3). The error bars signify the standard deviation across these replicates. Groups labeled with distinct letters are statistically significant from one another ($p < 0.05$), denoting meaningful differences.

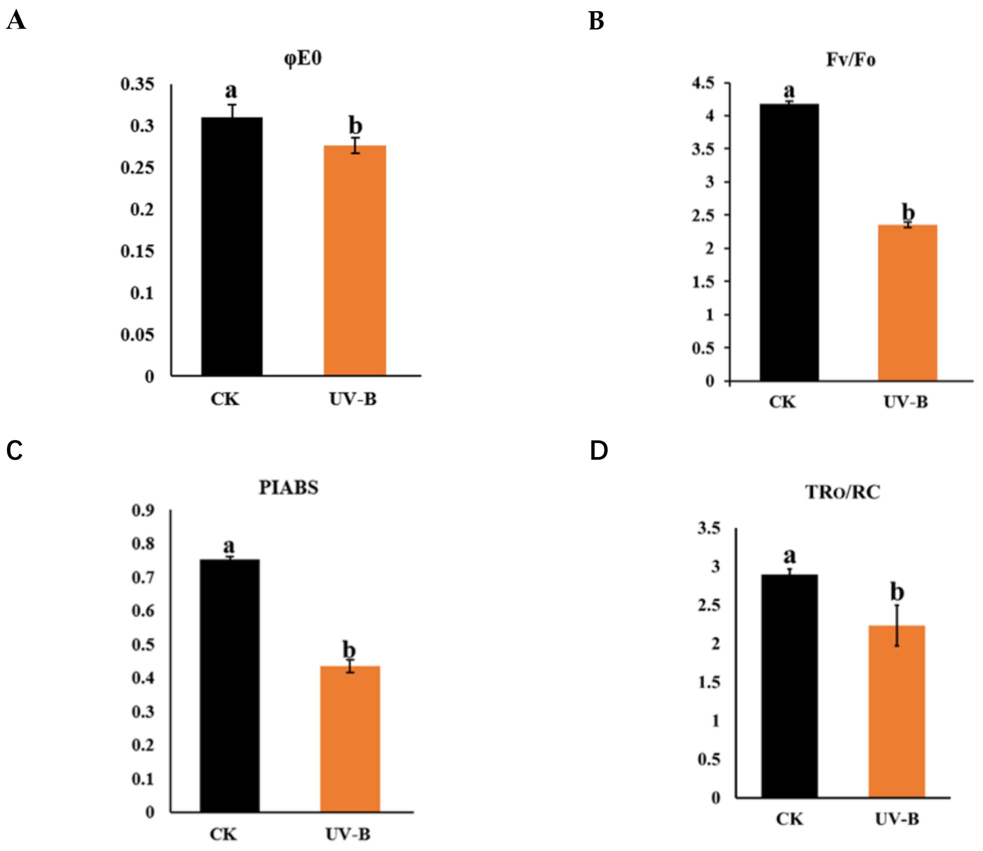

Supplementary Figure S2. Effect of UV-B radiation on the main measured parameters of the JIP of the *R. chrysanthum*. (A-D) The bar graph of φEo、Fv/Fo、PIABS and TRo/RC. The bar chart displays the average heights for each group, derived from three biological replicates (n = 3). The error bars signify the standard deviation across these replicates. Groups labeled with distinct letters are statistically significant from one another ($p < 0.05$), denoting meaningful differences.

Note: Some of the results in Supplementary Figures refer to previous studies with minor modifications[1,2].

1. Zhou, X.; Yu, W.; Gong, F.; Xu, H.; Lyu, J.; Zhou, X. Golden 2-like Transcription Factors Regulate Photosynthesis under UV-B Stress by Regulating the Calvin Cycle. *Plants (Basel)* **2024**, *13*, doi:10.3390/plants13131856.
2. Gong, F.; Zhou, X.; Yu, W.; Xu, H.; Zhou, X. Carotenoid Accumulation in the Rhododendron chrysanthum Is Mediated by Abscisic Acid Production Driven by UV-B Stress. *Plants* **2024**, *13*, doi:10.3390/plants13081062.
